# Supplementary material for: Targeting ACE2 with a camelid antibody inhibits SARS-CoV-2 binding and has protective effects in vivo
Source: Nat Commun. 2025 Nov 21;16:10268. doi: 10.1038/s41467-025-65144-w (PMC12638815; doi:10.1038/s41467-025-65144-w)
Supplement: Supplementary file 1 — Supplementary information [file 41467_2025_65144_MOESM1_ESM.pdf]

Supplementary Information

Targeting ACE2 with a camelid antibody inhibits SARS-CoV-2 binding and has protective effects *in vivo*

Simon Blachier[1], Marie-Christine Vaney[2], Laurine Conquet[3,4]†, Isabelle Staropoli[5]†, Ignacio Fernández[2], Emilie Giraud[6], Atousa Arbabian[2], Vincent Michel[7], Fruzsina Szilagyi[1], Salomé Guez[6], Alix Boucharlat[6], Jeanne Chiaravalli[6], Jaouen Tran-Rajau[6], Evelyne Dufour[8], Ahmed Haouz[9], Stéphane Petres[8], Delphine Planas[5,10], Xavier Montagutelli[3], Fabrice Agou[6], Pierre Lafaye[11], Gabriel Ayme[11]†, Olivier Schwartz[5,10]†, Felix A. Rey[2]†, Jost Enninga[1]†, Anne Brelot[1*]

Supplementary Figures

Figures

1. Sequence alignment of B07, B09, and B10 VHHs.
2. VHH binding on sACE2 and schematic of the binding competition assay.
3. VHHs binding on cells expressing ACE2.
4. Interactions between hACE2 and VHH B10.
5. Experimental 2|Fo|-|Fc| electron density maps at the site of interactions between hACE2 and VHHs.
6. Sequence alignment of human, hamster, and mouse ACE2.
7. Interactions of hACE2 with SARS-CoV-2 RBD (PDB: 6M0J) and footprints of interactions in different complexes.
8. Impact of hACE2 substitution on VHH binding.
9. B07-Fc activities.
10. Relative cell surface expression of ACE2 mutants and impact of substitutions on B07-Fc binding.
11. Separate color (top) or black and white (bottom) images of Fig. 7d.
12. Effect of VHH B07-Fc on hamsters.

Tables

1. Statistical analyses and IC50 related to Fig. 4a and 6g (S-Fuse assay), and 6h (N detection).
2. Crystallization conditions, data collection, and refinement statistics.
3. Van der Waals and polar interactions between hACE2 and VHH B10.
4. Van der Waals and polar interactions between hACE2 and VHH B07.
5. Van der Waals contacts between hACE2 and VHH B07 and between hACE2 and RBDs for a selection of viruses using hACE2 as a receptor.

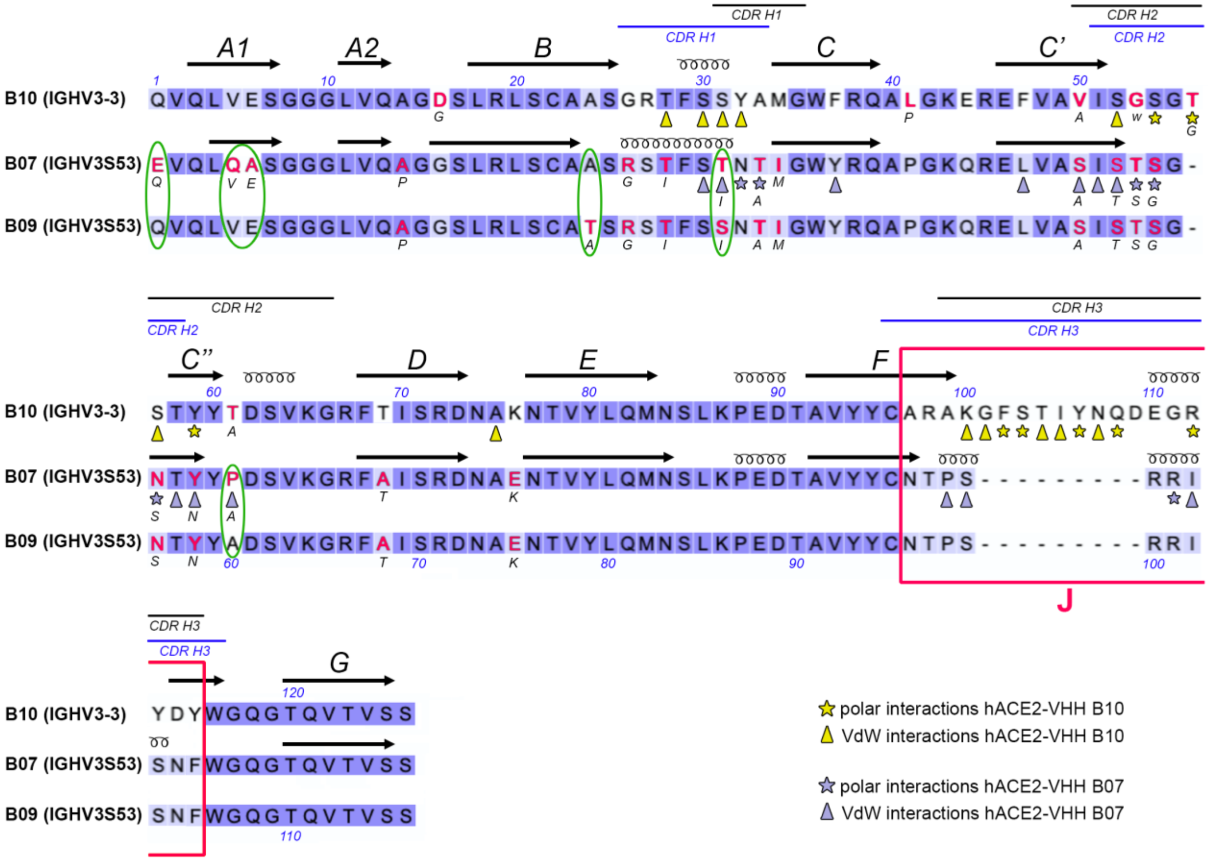

Supplementary Fig. 1: **Sequence alignment of B07, B09, and B10 VHHs.** In dark blue background are the residues strictly conserved; in light blue background are the residues conserved for 2 sequences; in white background, residues which are not conserved. Lines above the sequences mark the CDRs in the IMGT definition (in blue) and in the KABAT definition (in black). The sequence numbering, above and under the sequences, is according to IMGT definition and follow the numbering of the structures, respectively for B10 and B07. The contacts of B10 and B07 with hACE2 are reported under each residue as defined by the symbols in the inset. The somatic mutations are in red with germline residues in smaller font underneath. The secondary structure elements of B10 and B07 structures are indicated above each sequence. The green ovals indicate the amino acid difference between B07 and B09. J is the junction segment.

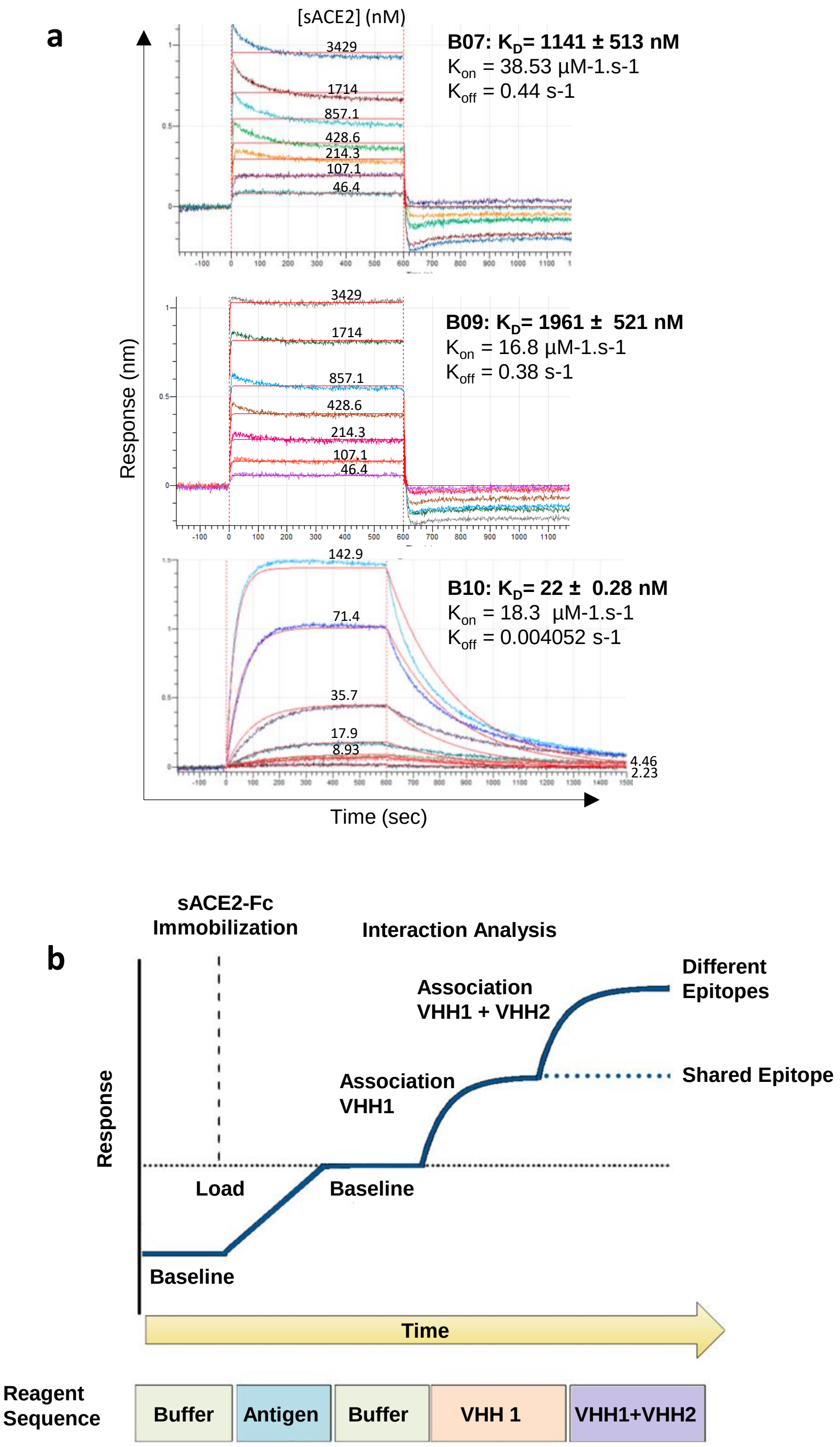

Supplementary Fig. 2: **VHH binding on sACE2 and schematic of the binding competition assay. a** Kinetic analysis of sACE2 on B07-His, B09-His, and B10-His VHHs by BioLayer Interferometry (BLI) using various concentrations of sACE2 (as indicated on the figure). Ni-NTA biosensors were used to immobilize VHH-His. K_D of each VHHs is indicated. One representative experiment out of three. Source Data are provided as a Source Data file. **b** Schematic representation of the binding competition assay. Following a baseline step, the sACE2-Fc (5 µg/mL) was immobilized onto the AHC biosensors. After a second baseline step, a first VHH was applied (VHH1; 5 µg/mL). The sensor was dipped in a mixture of VHH1 + the competitor VHH2 at the same concentration (5 µg/mL).

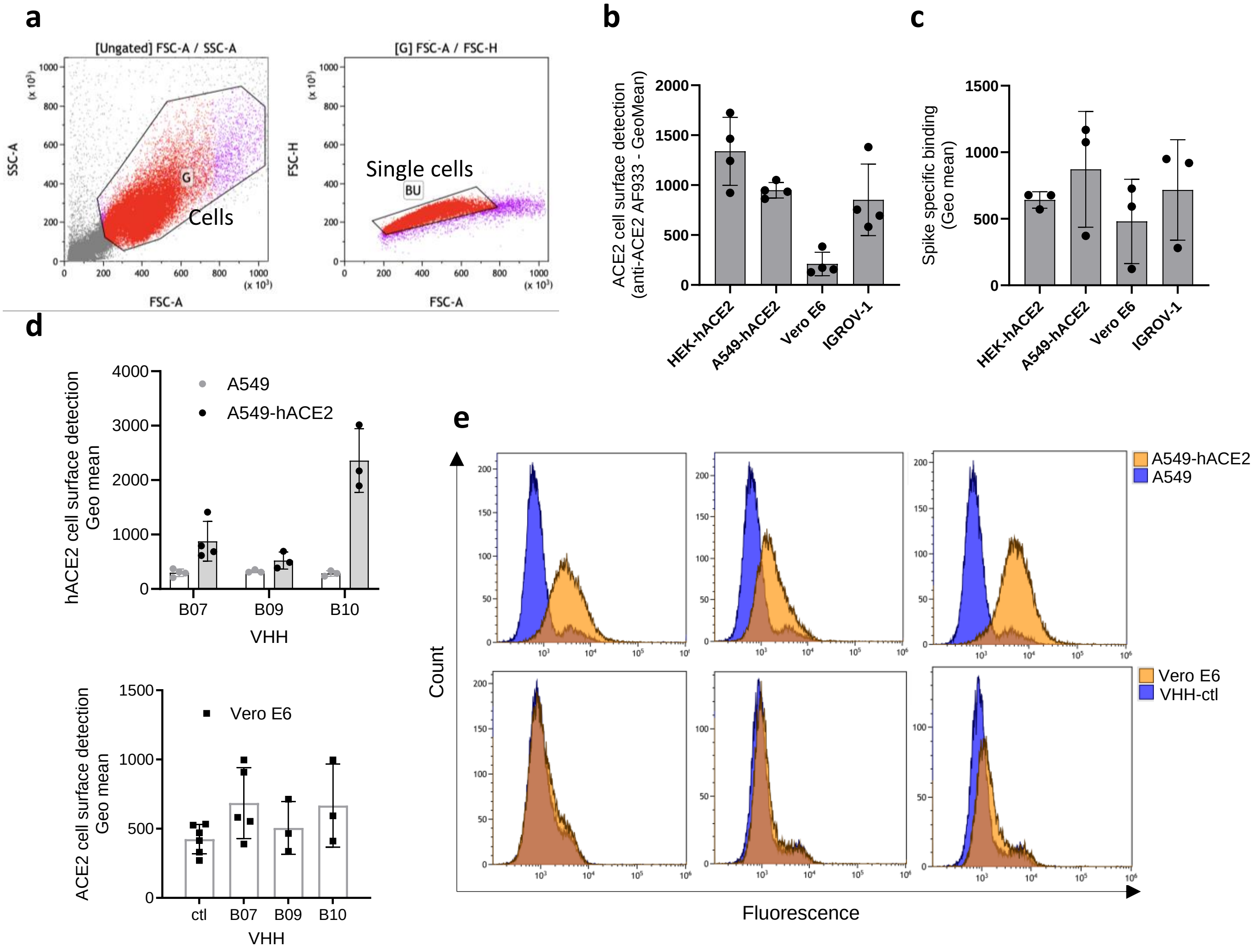

Supplementary Fig. 3: VHHs binding on cells expressing ACE2. **a** Example of FACS sequential gating strategy for detection of hACE2 on cell surface of HEK293-hACE2 cells by VHH or mAbs. **b** ACE2 cell surface detection by the commercial anti-ACE2 antibody AF933 (R&D system, 1 µg/mL). Data are mean ± SD of 4 independent experiments. **c** Spike binding to ACE2-expressing cells. Cells were incubated with soluble spike (S) protein (ancestral spike) (10 µg/mL) and stained with an anti-S antibody. Data are mean ± SD of 3 independent experiments. **d** B07, B09, and B10 binding on A549-hACE2 (top) and Vero E6 (bottom) cells. Cells were incubated with the VHHs (10 µg/mL), stained with an anti-myc antibody and a AF488-conjugated anti-mouse antibody, before being analyzed by flow cytometry. Parental A549 cells or myc-tag VHH IgE were used as control. Data are mean ± SD of 3 (B09, B10;); 4 (A549/ B07), or 5 (Vero E6/B07) independent experiments. **e** Fluorescence diagram overlays: B07, B09, B10 efficacy on different cell lines expressing exogenous (A549-hACE2) or endogenous non-human ACE2 (Vero E6). Background (blue) corresponds to the fluorescence intensity obtained on parental cells (A549) or using a VHH control (anti-IgE VHH) (Vero E6). Source Data are provided as a Source Data file.

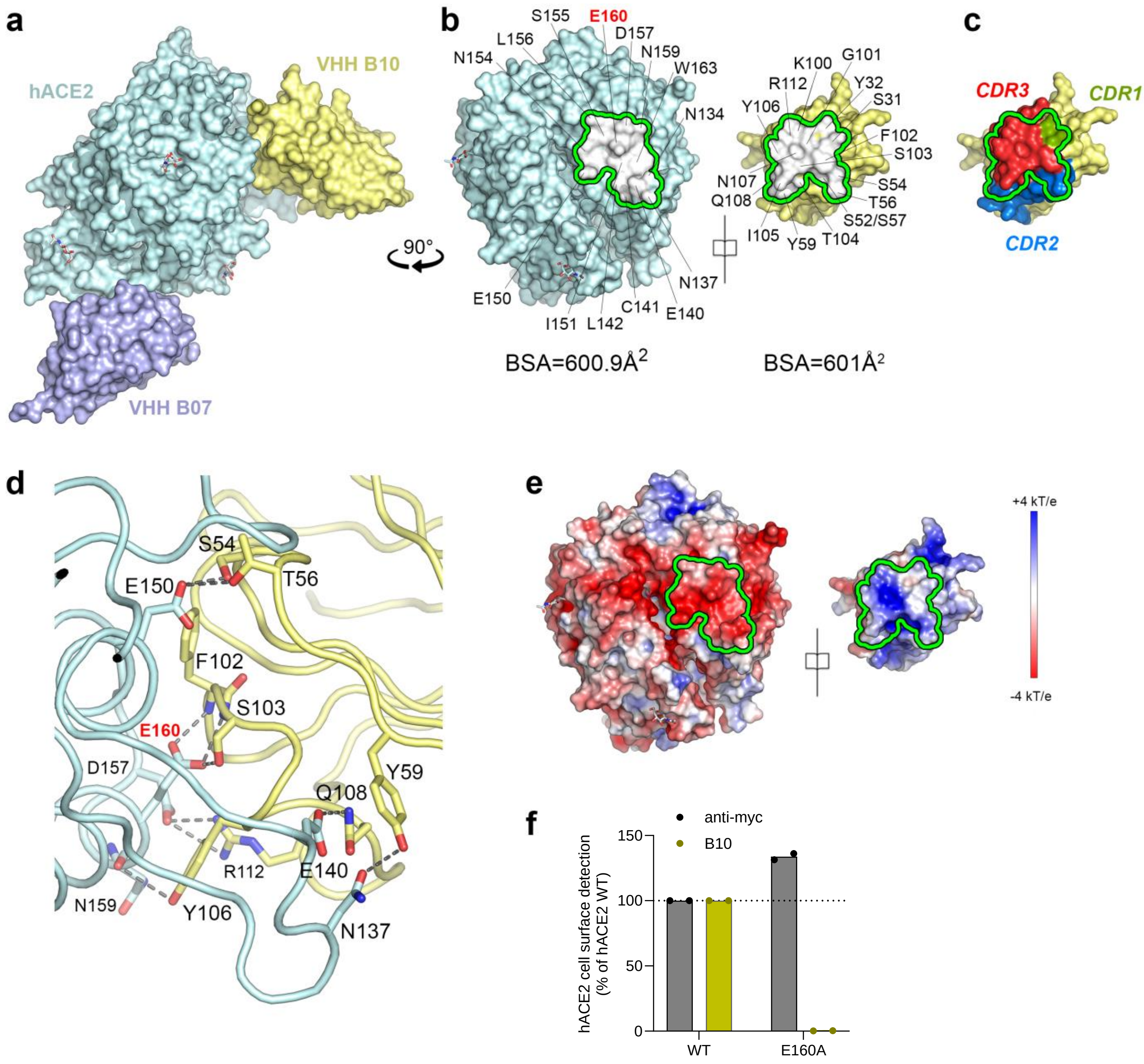

Supplementary Fig. 4: Interactions between hACE2 and VHH B10. a Structure of hACE2/B07/ B10 complex. **b** Rotated view from (a) as indicated and open-book representation of complex hACE2 and VHH B10 (for clarity the VHH B07 was not displayed). The surface of contacts between hAEC2 and VHH B10 are displayed in white and contoured in green. The buried surfaces (BSA) are indicated under each surface. **c** Open-book interactions as in (b) with B10 colored complementarity determining regions (CDRs) in green (CDR1), blue (CDR2), and red (CDR3). **d** Detailed interaction between hACE2 and B10 (hydrogen bonds in black dashed lines). **e** Electrostatic potential mapped on the surface of the structure of hACE2 and B10. **f** Impact of hACE2 E160A substitution on B10 binding. Cells were transfected with myc tagged or untagged hACE2 E160A (and eGFP), incubated with an anti-myc antibody or B10 (10 µg/mL) and stained with a mouse anti-myc antibody and a AF647-conjugated anti-mouse antibody before being analyzed by flow cytometry. Data are mean of two independent experiments. Source Data are provided as a Source Data file.

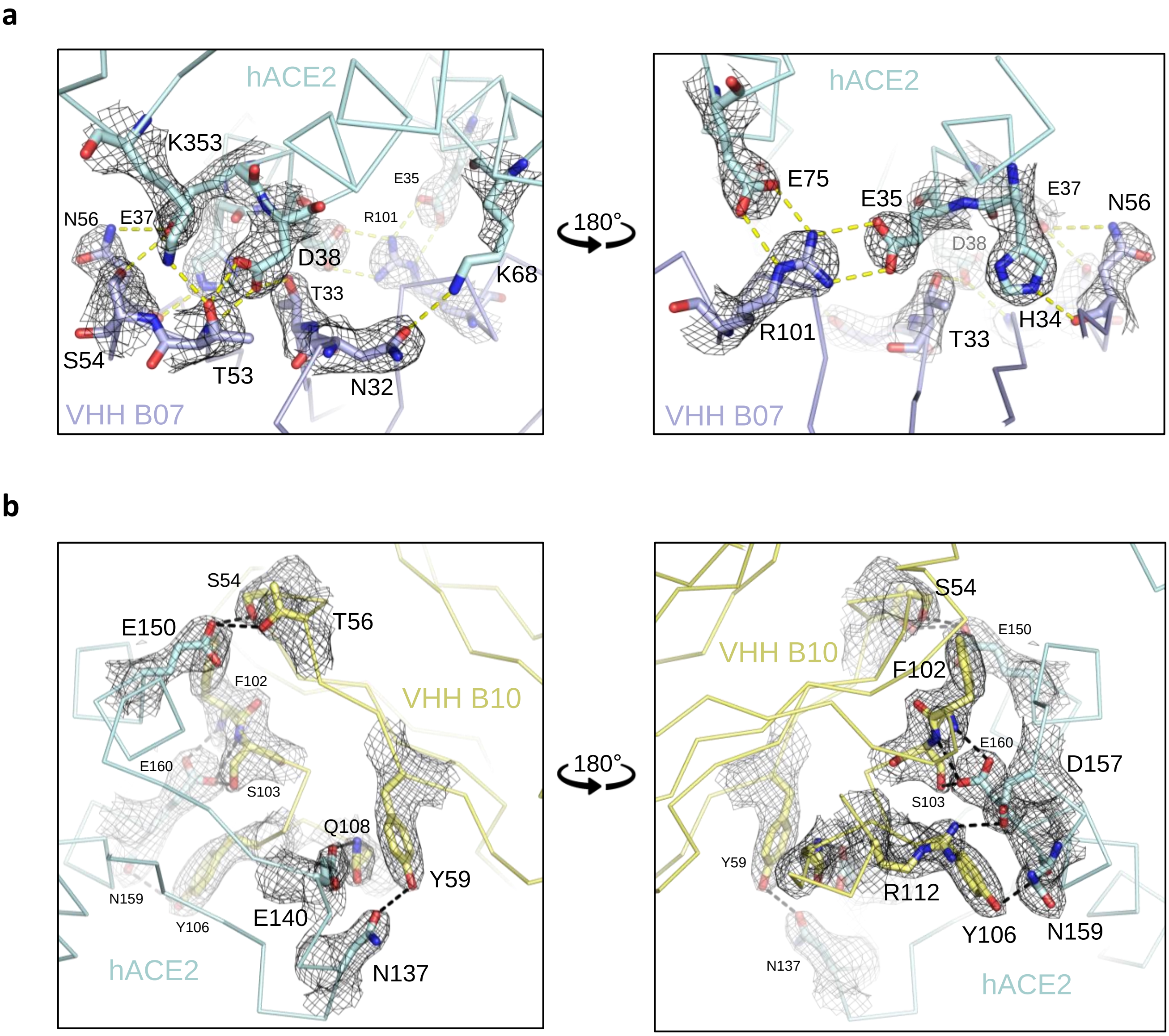

Supplementary Fig. 5: Experimental 2|Fo|-|Fc| electron density maps at the site of interactions between hACE2 and VHHs. For clarity, only the density of the interacting residues, shown as color-coded sticks, is displayed. The maps are contoured at 3 sigma. **a** Two views at 180° each, showing the densities at the site of interactions between hACE2 and VHH B07. **b** Two views at 180° each, showing the densities at the site of interactions between hACE2 and VHH B10.

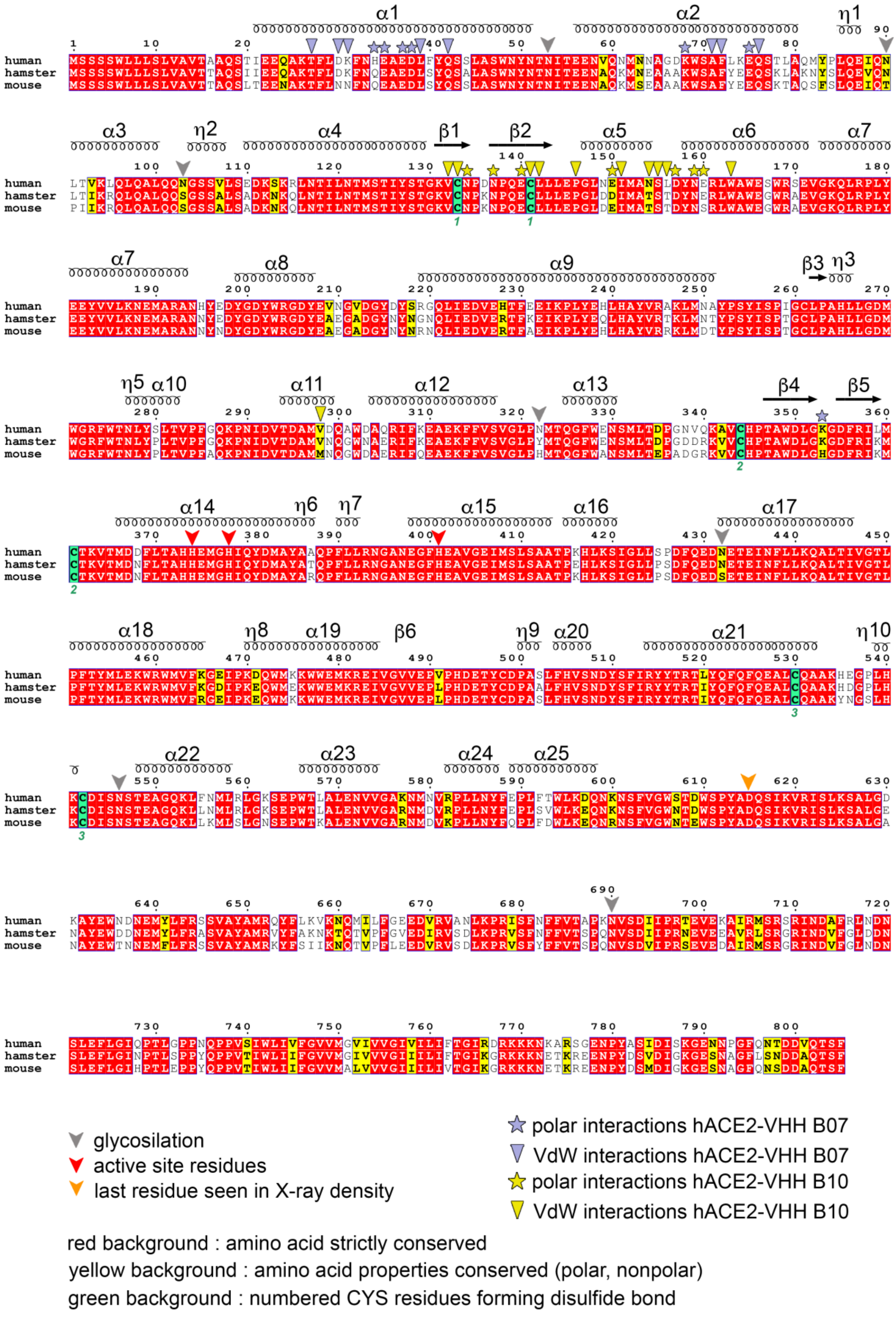

Supplementary Fig. 6: **Sequence alignment of human, hamster, and mouse ACE2.**

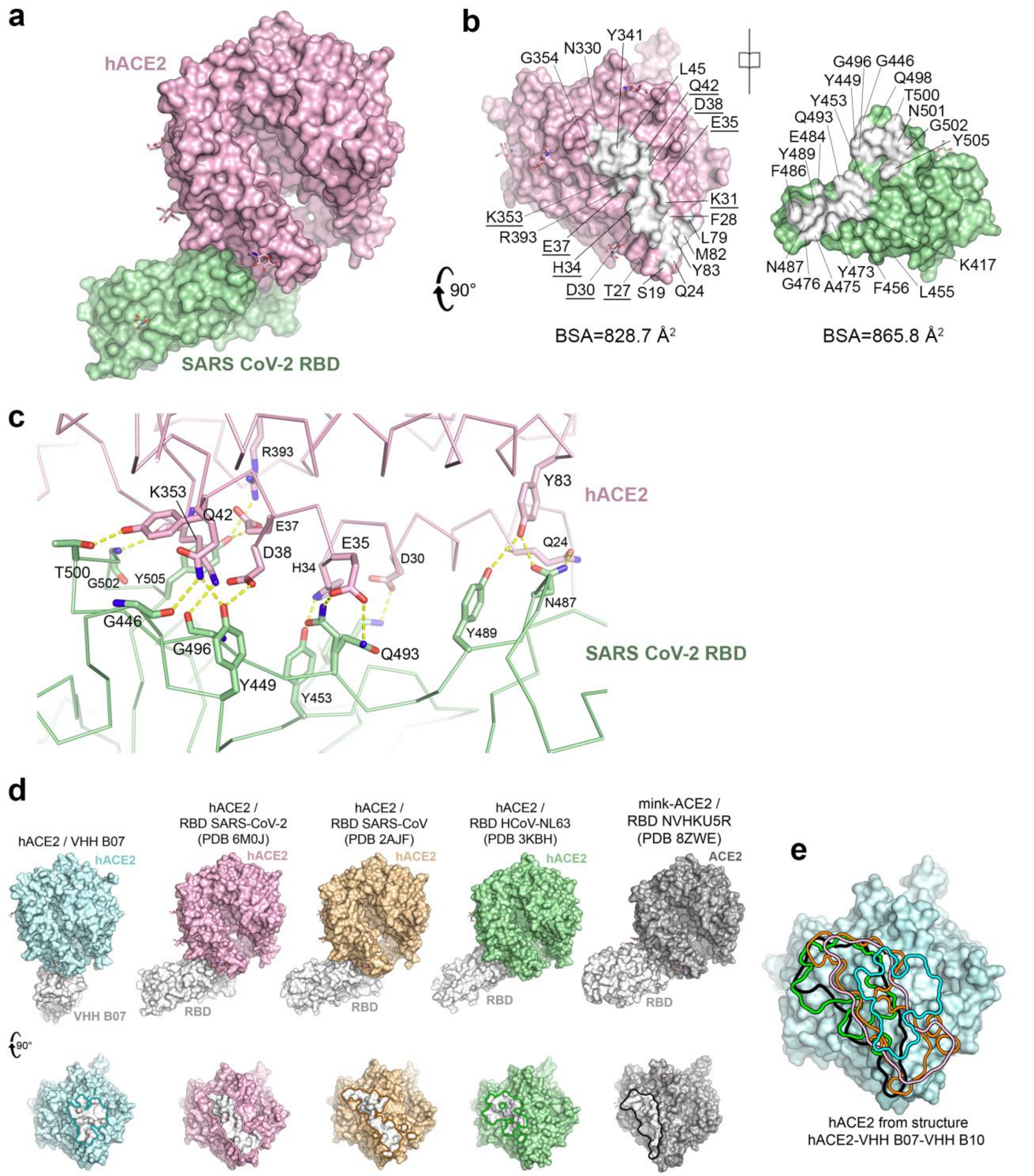

Supplementary Fig. 7: Interactions of hACE2 with SARS-CoV-2 RBD (PDB: 6M0J) and footprints of interactions in different complexes. **a** Structure of hACE2 (pink) / RBD SARS-CoV-2 (green) (PDB 6M0J) displayed as surfaces. **b** Open-book representation and footprints (in white) of RBD on hACE2 (left) and hACE2 on RBD (right). The buried surface (BSA) are indicated under each surface. The residues in hACE2 also involved in the interactions with VHH B07 are underlined. **c** Detailed interactions between hACE2 and RBD (hydrogen bonds in yellow dashed lines). **d** Footprints of VHH B07 and RBDs on ACE2 for a selection of viruses. Top, structures of complexes as indicated. Bottom, 90° views from top, in which the footprints (in white) of each molecule (VHH B07 and RBDs) on the top views are displayed on ACE2. **e** Each footprint is represented as an outline colored according to (d) and superposed on the hACE2/VHH B07/VHH B10 structure showing their overlap.

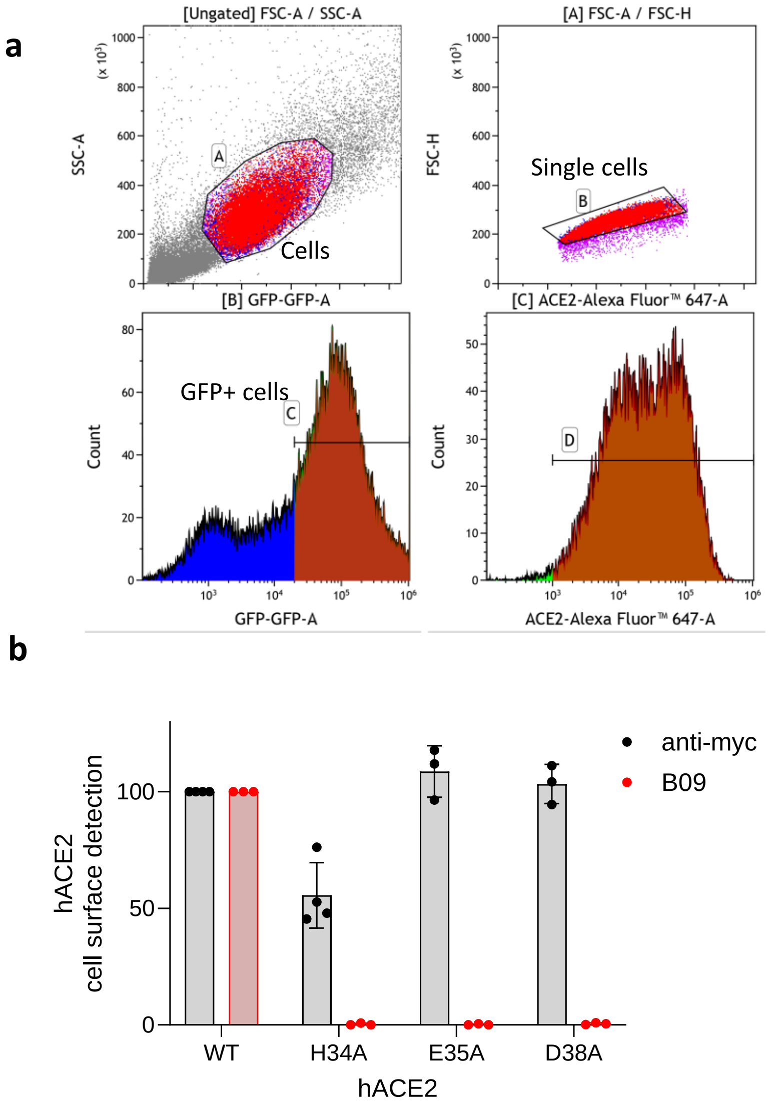

Supplementary Fig. 8: **Impact of hACE2 substitution on VHH binding. a** Example of FACS sequential gating strategy for B07 or anti-myc detection of hACE2 at the cell surface of HEK293 cells co-transfected with plasmids coding hACE2 and eGFP (ratio 5/1, respectively). **b** Impact of alanine substitution on B09 binding. Cells were transfected with untagged or myc tag hACE2 alanine mutants (and eGFP), incubated with an anti-myc antibody or B09 (10 µg/mL) and stained with a mouse anti-myc antibody and a AF647-conjugated anti-mouse antibody before being analyzed by flow cytometry. Data are mean ± SD of three (E35A, D38A) or four (H34A) independent experiments. Source Data are provided as a Source Data file.

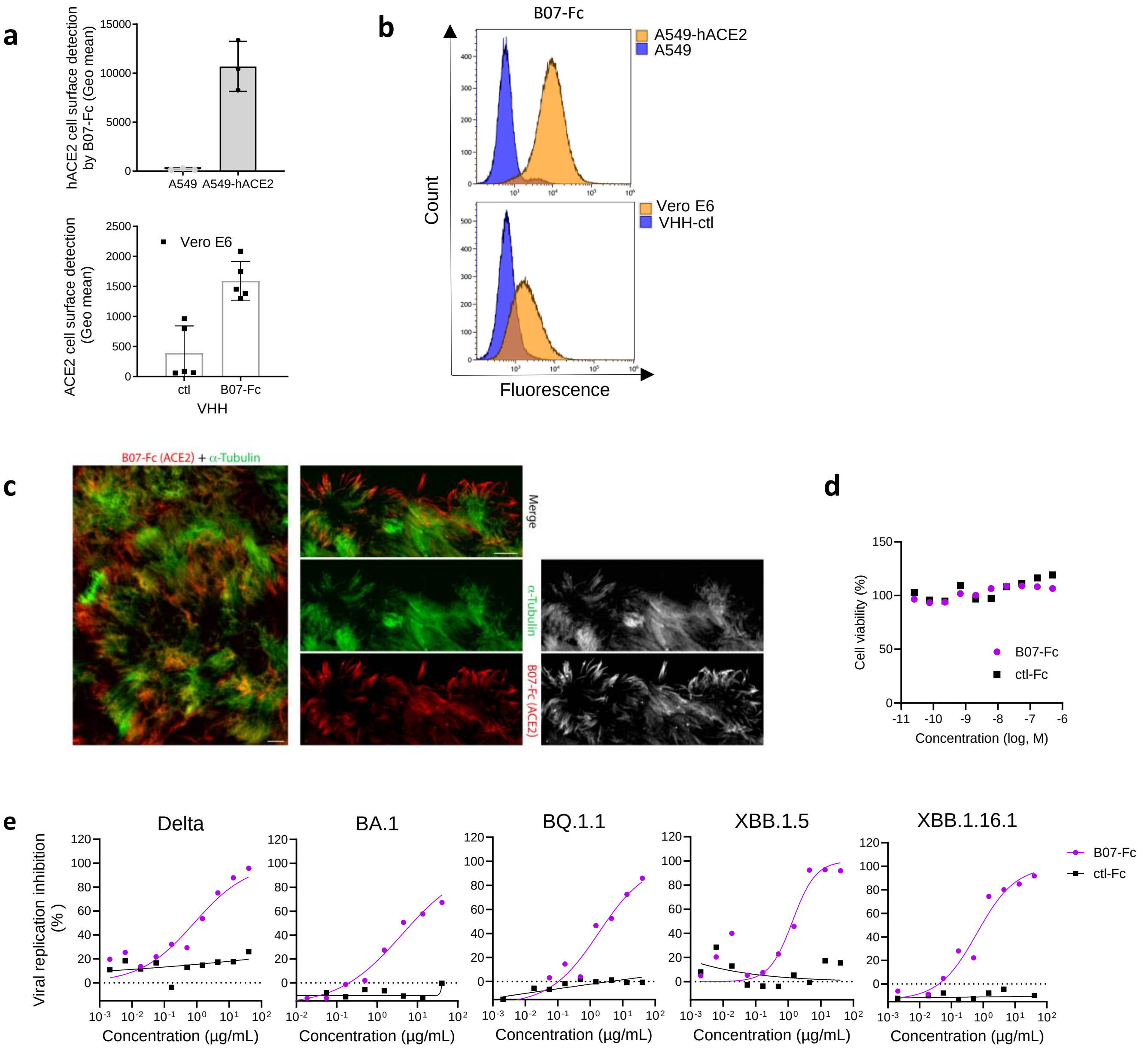

Supplementary Fig. 9: **B07-Fc activities**. **a** B07-Fc binding on A549-hACE2 and Vero E6 cells. Cells were incubated with 0.1 µg/mL B07-Fc and a AF488-conjugated secondary antibody, and analyzed as in Fig 6b. Data are mean ± SD of 3 (A549) or 5 (Vero E6) independent experiments. **b** Fluorescence diagram overlays performed as in Fig. 6c on A549-hACE2 and Vero E6 cells. **c** B07-Fc detection of hACE2 on ciliated cells of human nasal epithelial cells (hNEC). Representative immunofluorescence staining (out of 4 independent experiments) of hACE2 (Red: B07-Fc staining) in combination with α-tubulin (Green). Scale bars: left, 10 µm; right, 5 µm. **d** Vero E6 cells viability in the presence of B07-Fc measured by quantitating ATP using the CellTiter-Glo assay (mean of one experiment in triplicates). **e** Inhibition of SARS-CoV-2 replication by B07-Fc measured by RT-qPCR. Vero E6 cells pre-incubated with B07-Fc were infected with Delta B.1.617.2, BA.1, BQ.1.1, XBB.1.5 and XBB.1.16.1 (MOI and incubation time adjusted depending on virus replication, see Methods) and viral replication was measured by quantitative RT-qPCR (mean of one experiment in triplicates). Source Data are provided as a Source Data file.

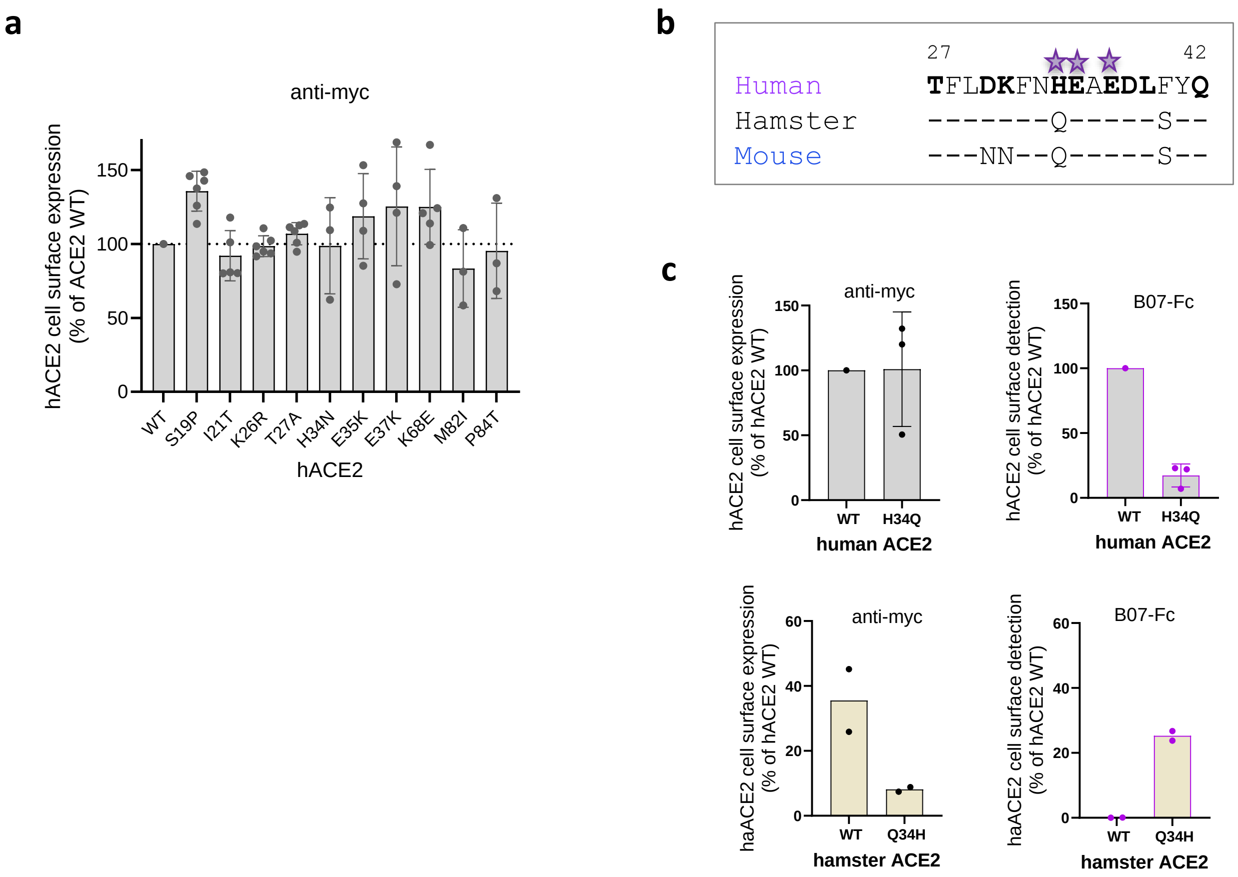

Supplementary Fig. 10: Relative cell surface expression of ACE2 mutants and impact of substitutions on B07-Fc binding. HEK293 cells transfected with plasmids encoding myc-hACE2-WT or the indicated mutant were stained with an antibody against the myc epitope or with B07-Fc and analyzed by flow cytometry. **a** Bars represent staining efficiency for cells expressing myc-hACE2 mutants relative to cells expressing myc-hACE2-WT after anti-myc staining. Data are means ± SD of three (H34N, M82I, P84T), four (E35K, E37K), five (I21T, K68E), or six (S19P, K26R, T27A) independent experiments. **b** Alignment of the amino acid sequences of human, hamster, and mouse ACE2 from position 27 to 42. Residues involved in B07 epitope are in bold. Purple stars indicate polar interactions between hACE2 and B07. **c** Bars represent HEK293 cells transfected with plasmids encoding myc-hACE2 (top) or myc-hamster ACE2 (haACE2, bottom) and stained with an antibody against the myc epitope (left) or with B07-Fc (right) and analyzed by flow cytometry. Data are means ± SD of three independent experiments (human ACE2), or mean of 2 independent experiments (hamster ACE2). Source Data are provided as a Source Data file.

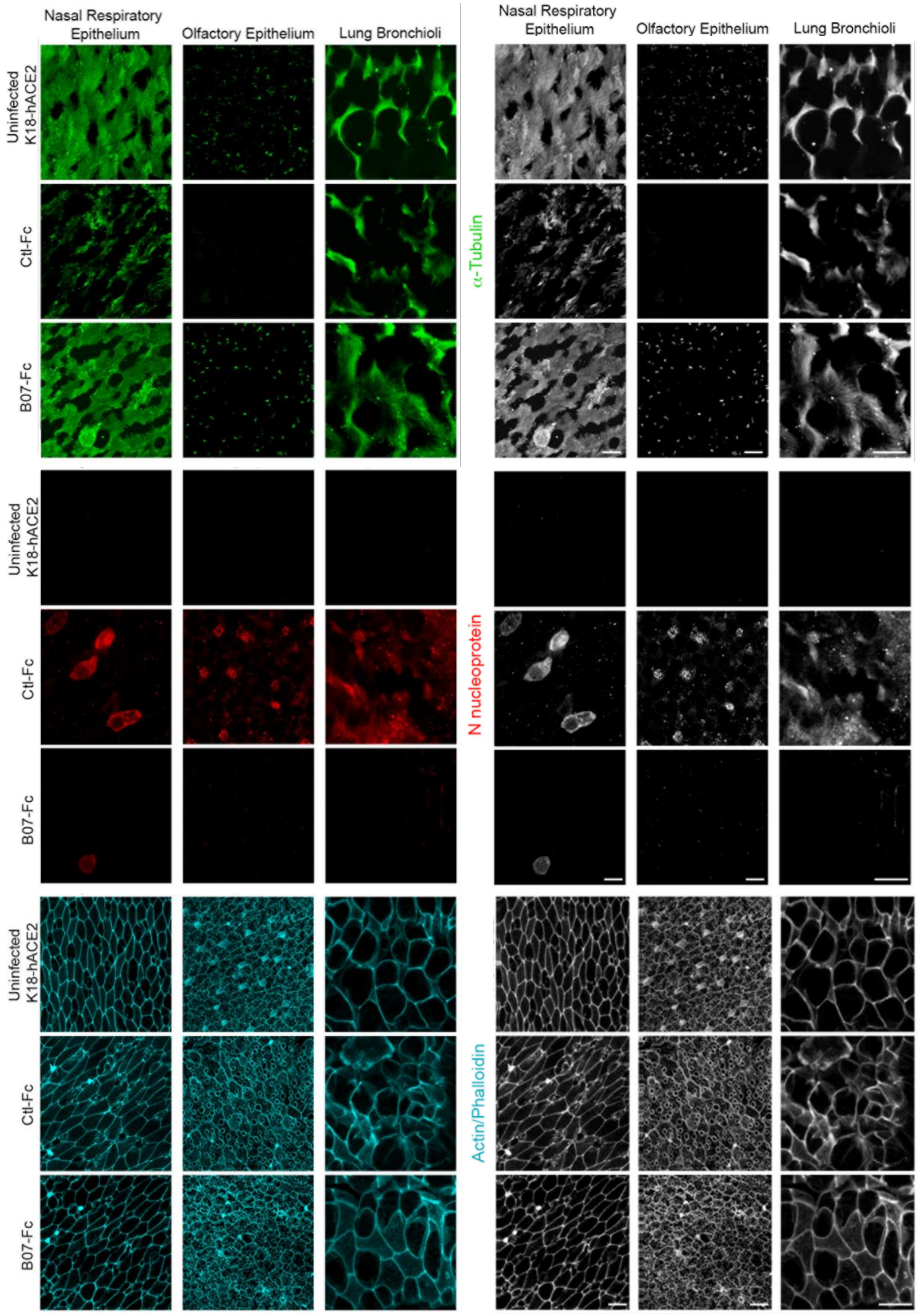

Supplementary Fig. 11: Separate color (top) or black and white (bottom) images of Fig. 7d. Phalloidin (F-actin), SARS-CoV-2 nucleocapsid (N) (viral replication), and tubulin staining on nasal respiratory epithelium, olfactory epithelium and lung bronchioli extracted from K18-hACE2 uninfected mice, and animals that received VHH-Fc (Ctl-Fc or B07-Fc). Representative immunofluorescence staining of phalloidin (Cyan), SARS-CoV-2 N protein (Red), and tubulin (Green). The staining was performed on nasal, olfactory, and pulmonary epithelium of 2 VHH ctl-treated mice and 4 B07-Fc treated mice. Scale bars: 10 µm.

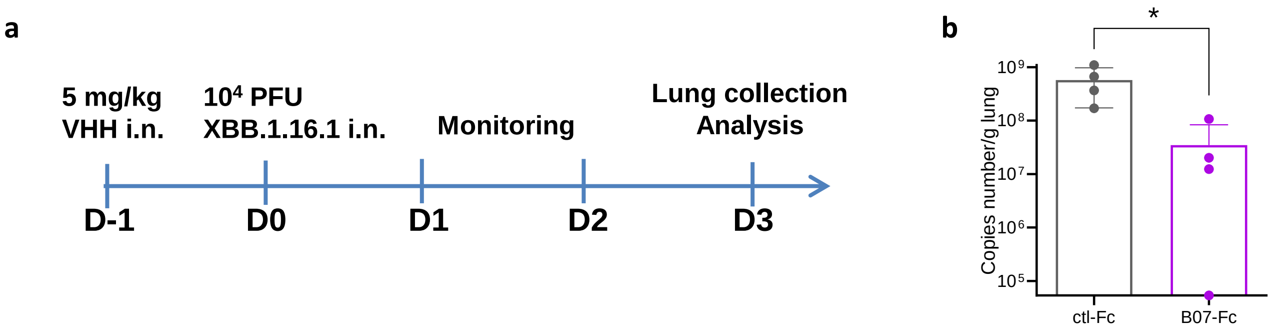

Supplementary Fig. 12: Effect of VHH B07-Fc on hamsters. a Schematic diagram showing the experimental design of B07-Fc prophylaxis in XBB.1.16.1 infected hamsters. Animals received intranasally (i.n.) 5 mg/kg VHH-B07-Fc (B07-Fc) or 5 mg/kg VHH-Fc ctl (ctl-Fc). Twenty four hours later, they were infected with 10^4 PFU XBB.1.16.1 intranasally (i.n.). Three days post-infection, lungs were collected for analysis. Animal behavior and weight were followed each day. **b** RNA load measured by RT-qPCR of SARS-CoV-2 in lung. Data are mean ± SD of 4 animals. Mann-Witney test (two-tailed): P value 0.0286*. Source Data are provided as a Source Data file.

a

B07	P values CI (µg/mL)	R^2
Delta	16.97 to 51.41	0.8712
BA.1	8.563 to 23.63	0.8616
BQ.1.1	3.885 to 6.914	0.9890
XBB1.5	6.116 to 13.25	0.9192
XBB.1.16.1	4.261 to 6.938	0.9909
EG.5.1.3	2.628 to 3.891	0.9948
BA.2.86.1	0.7223 to 2.903	0.9412

B09	P values CI (µg/mL)	R^2
Delta	12.39 to 26.22	0.8901
BA.1	17.52 to 41.94	0.9223
BQ.1.1	4.839 to 12.49	0.9711
XBB1.5	6.435 to 10.67	0.9669
XBB.1.16.1	8.562 to 16.19	0.9815
EG.5.1.3	4.643 to 12.95	0.9631
BA.2.86.1	1.300 to 8.622	0.8942

b

VHH	Delta	BA.1	BQ.1.1	XBB.1.5	XBB.1.16.1	EG.5.1.3	BA.2.86.1
B07	1937.2	932.8	339.8	590.4	356.9	209.9	95.1
B09	1180.7	1776.3	509.1	543.2	771.2	507.8	219.5
B10	-	-	-	-	-	-	-
Ctl	-	-	-	-	-	-	-

IC50 (nM)

c

S-Fuse	p-values CI (µg/mL)	R^2
Delta	0.1714 to 0.3072	0.9702
BA.1	0.1603 to 0.2168	0.9891
BQ.1.1	0.09368 to 0.1397	0.9761
XBB1.5	0.07860 to 0.1756	0.9420
XBB.1.16.1	0.08340 to 0.1088	0.9908
EG.5.1.3	0.06759 to 0.08863	0.9923
BA.2.86.1	0.07032 to 0.1454	0.9546
JN.1.1	0.1222 to 0.1561	0.9862
KP3.3	0.2897 to 0.3875	0.9801

Anti-N	p-values CI (ng/mL)	R^2
JN.1.1	3.71 to 5.50	0.9928
KP.3.3	5.45 to 6.56	0.9966

Supplementary Table 1: Statistical analyses and IC50 related to Fig. 4a and 6g (S-Fuse assay), and 6h (N detection). a P-values 95% confidence intervals for VHH IC50 values and R^2 values relative to B07 and B09 IC50s (Fig. 4a). Curves were fitted with a non-linear regression model (variable slope). **b** B07 IC50 values in nM (Fig. 4a). **c** P-values 95 % confidence intervals and R^2 values relative to B07-Fc IC50 obtained in S-Fuse (Fig. 6g) or inhibition infection (anti-N) assays (Fig. 6h). Curves were fitted with a non-linear regression model (variable slope). Source Data are provided as a Source Data file.

	hACE2 - VHH B07 - VHH B10
**Data collection**[£]	
Space group	P 1 2_1 1
Cell dimensions	
a, b, c (Å)	97.9, 108.1, 146.1
α, β, γ (°)	90, 98.9, 90
High resolution (Å) cut-off at $CC_{1/2}$>30%	2.69
Resolution (Å)	48.37-2.69 (2.76-2.69)
Rmerge (all I+ & I-) (%)	23.6 (159)
Rmeas (all I+ & I-) (%)	25.5 (171.5)
Rpim (all I+ & I-) (%)	9.5 (63.6)
Measured reflections	590,491 (43,028)
Unique reflections	83,613 (6,015)
<I/σI>	9.2 (1.3)
Completeness (%)	99.8 (97)
Multiplicity	7.1 (7.2)
$CC_{1/2}$ (%)	99.2 (37.5)
B-Wilson (Å^2)	63.8
Number of molecules/asymmetric unit	3x(ACE2 - VHH B07 - VHH B10)
**Refinement**[£]	
Resolution	48.3-2.69 (2.71-2.69)
Number of reflections Work/Free	83,594 (1,589) / 4,180 (83)
Rwork (%) / Rfree (%)	21.9 (34.1) / 25.4 (39.1)
Root mean square deviation	
Bond lengths (Å)	0.007
Bond angles (°)	0.87
No. of atoms	
Protein	20,123
Solvent	104
Chloride ions	4
Glycans	13
B-mean (Å^2) (all atoms)	70.2
Ramachandran (%)[$]	
Favoured / Allowed / Outliers	97 / 2.88 / 0.12

[£] Values in parentheses are for the highest-resoluion shell
[$] Ramachandran statistics were calculated with MolProbity

Supplementary Table 2: **Crystallization conditions, data collection and refinement statistics.**

Residues in hACE2 and VHH B10 involved in van der Waals contacts

hACE2	ss	H/S	ASA ($Å^2$)	BSA ($Å^2$)	ΔiG (kcal/mol)
VAL 132	β1		1.74	1.23 ||||||||	-0.01
CYS 133	β1		37.99	1.28		0.02
ASN 134	β1		59.59	27.51 |||||	-0.23
ASN 137	β2	H	61.75	16.45 |||	-0.28
GLU 140	β2	H	103.32	79.65 ||||||||	-0.41
CYS 141	β2		37.18	3.08		0.05
LEU 142	β2		57.57	32.86 ||||||	0.52
PRO 146	loop β2-α5		83.77	2.68		0.04
GLU 150	α5	H	111.20	74.53 |||||||	-0.51
ILE 151	α5		25.26	24.42 ||||||||||	0.39
ASN 154	α5		110.53	43.86 ||||	0.29
SER 155	loop α5-α6		14.63	12.32 |||||||||	0.15
LEU 156	loop α5-α6		122.74	70.82 ||||||	1.01
ASP 157	loop α5-α6	HS	71.03	54.04 ||||||||	0.09
ASN 159	α6	H	89.83	23.87 |||	0.24
GLU 160	α6	H	75.85	75.85 |||||||||||	-0.26
TRP 163	α6		107.99	51.76 |||||	0.81
VAL 298	α11		93.84	4.66		0.07

VHH B10	CDR	H/S	ASA ($Å^2$)	BSA ($Å^2$)	ΔiG (kcal/mol)
THR 28	FR1		72.00	6.86		0.11
SER 30	FR1		55.90	1.47		-0.02
SER 31	CDR1		53.24	34.54 |||||||	0.28
TYR 32	CDR1		55.51	23.51 |||||	0.04
SER 52	CDR2		4.26	2.25 ||||||	0.03
SER 54	CDR2	H	54.21	10.68 ||	0.08
THR 56	CDR2	H	125.88	16.31 ||	-0.14
SER 57	CDR2		48.79	10.31 |||	-0.00
TYR 59	CDR2	H	98.43	39.18 ||||	0.28
ALA 75	FR3		98.79	4.67		0.07
LYS 100	CDR3		92.82	28.85 ||||	0.07
GLY 101	CDR3		21.23	21.06 ||||||||||	0.34
PHE 102	CDR3	H	98.61	95.00 ||||||||||	1.38
SER 103	CDR3	H	36.15	29.79 |||||||||	0.33
THR 104	CDR3		14.96	4.54 ||||	-0.05
ILE 105	CDR3		151.17	138.96 ||||||||||	1.99
TYR 106	CDR3	H	151.51	89.10 ||||||	0.91
ASN 107	CDR3		0.62	0.62 |||||||||||	-0.01
GLN 108	CDR3	H	58.04	16.87 |||	-0.28
ARG 112	CDR3	HS	54.59	26.41 |||||	-0.67

Polar contacts between hACE2 and VHH B10

hACE2				VHH B10	
atom	ss	distance (Å)		atom	CDR
ASN 137 OD1	β2	H	3.61	TYR 59 OH	CDR2
GLU 140 OE2	β2	H	3.40	GLN 108 NE2	CDR3
GLU 150 OE2	α5	H	2.83	SER 54 OG	CDR2
GLU 150 OE2	α5	H	3.36	THR 56 OG1	CDR2
ASP 157 OD2	α6	HS	3.63	ARG 112 NH1	FR4
ASP 157 OD2	α6	HS	2.93	ARG 112 NH2	FR4
ASN 159 O	α6	H	3.57	TYR 106 OH	CDR2
GLU 160 OE1	α6	H	2.58	SER 103 OG	CDR3
GLU 160 OE1	α6	H	3.10	SER 103 N	CDR3
GLU 160 OE2	α6	H	2.88	PHE 102 N	CDR2

Supplementary Table 3: **Van der Waals and polar interactions between hACE2 and VHH B10**. CDR: complementary-determining region (KABAT definition); FR: Framework region; ss: secondary structure in hACE2; H: hydrogen bonds; S: salt-bridges; ASA: accessible surface area; BSA: buried surface area; ∆Gi: solvation energy effect; Vertical bars in BSA columns indicate the buried area percentage (with one bar per 10% of ASA).

Residues in hACE2 and VHH B07 involved in van der Waals contacts

<table>
<tr><th>hACE2</th><th>SS</th><th>HS</th><th>ASA (Å^2)</th><th>BSA (Å^2)</th><th>ΔiG (kcal/mol)</th></tr>
<tr><td>THR 27</td><td>a1</td><td></td><td>71.69</td><td>13.25 ||</td><td>0.20</td></tr>
<tr><td>ASP 30</td><td>a1</td><td></td><td>99.65</td><td>18.60 ||</td><td>0.23</td></tr>
<tr><td>LYS 31</td><td>a1</td><td></td><td>121.90</td><td>48.19 ||||</td><td>0.77</td></tr>
<tr><td>HIS 34</td><td>a1</td><td>H</td><td>139.56</td><td>120.57 |||||||||</td><td>0.60</td></tr>
<tr><td>GLU 35</td><td>a1</td><td>HS</td><td>85.73</td><td>79.49 ||||||||||</td><td>-0.32</td></tr>
<tr><td>GLU 37</td><td>a1</td><td>H</td><td>81.81</td><td>46.14 ||||||</td><td>-0.54</td></tr>
<tr><td>ASP 38</td><td>a1</td><td>H</td><td>80.63</td><td>77.51 ||||||||||</td><td>-0.25</td></tr>
<tr><td>LEU 39</td><td>a1</td><td></td><td>44.52</td><td>33.13 ||||||||</td><td>0.53</td></tr>
<tr><td>GLN 42</td><td>a1</td><td></td><td>94.45</td><td>48.95 ||||||</td><td>-0.11</td></tr>
<tr><td>LYS 68</td><td>a2</td><td>H</td><td>121.21</td><td>51.24 |||||</td><td>-0.91</td></tr>
<tr><td>ALA 71</td><td>a2</td><td></td><td>62.37</td><td>25.40 |||||</td><td>0.31</td></tr>
<tr><td>PHE 72</td><td>a2</td><td></td><td>14.52</td><td>14.52 ||||||||||</td><td>0.21</td></tr>
<tr><td>GLU 75</td><td>a2</td><td>HS</td><td>94.37</td><td>54.58 ||||||</td><td>-0.29</td></tr>
<tr><td>GLN 76</td><td>a2</td><td></td><td>8.80</td><td>2.04 |||</td><td>-0.02</td></tr>
<tr><td>LYS 353</td><td>loop b4-b5</td><td>H</td><td>119.29</td><td>28.47 |||</td><td>-0.85</td></tr>
</table>

<table>
<tr><th>VHH B07</th><th>CDR</th><th>HS</th><th>ASA (Å^2)</th><th>BSA (Å^2)</th><th>ΔiG (kcal/mol)</th></tr>
<tr><td>SER 30</td><td>FR1</td><td></td><td>58.84</td><td>1.96 |</td><td>-0.02</td></tr>
<tr><td>THR 31</td><td>CDR1</td><td></td><td>101.13</td><td>52.93 ||||||</td><td>0.21</td></tr>
<tr><td>ASN 32</td><td>CDR1</td><td>H</td><td>40.87</td><td>21.48 ||||||</td><td>-0.26</td></tr>
<tr><td>THR 33</td><td>CDR1</td><td>H</td><td>50.34</td><td>47.92 ||||||||||</td><td>-0.09</td></tr>
<tr><td>TYR 37</td><td>FR2</td><td></td><td>8.16</td><td>1.11 ||</td><td>-0.01</td></tr>
<tr><td>LEU 47</td><td>FR2</td><td></td><td>55.19</td><td>25.76 |||||</td><td>0.41</td></tr>
<tr><td>SER 50</td><td>CDR2</td><td></td><td>17.02</td><td>16.04 ||||||||||</td><td>0.07</td></tr>
<tr><td>ILE 51</td><td>CDR2</td><td></td><td>11.20</td><td>0.33 |</td><td>0.01</td></tr>
<tr><td>SER 52</td><td>CDR2</td><td></td><td>29.38</td><td>29.38 ||||||||||</td><td>0.43</td></tr>
<tr><td>THR 53</td><td>CDR2</td><td>H</td><td>62.84</td><td>34.94 ||||||</td><td>-0.20</td></tr>
<tr><td>SER 54</td><td>CDR2</td><td>H</td><td>93.93</td><td>22.99 |||</td><td>0.24</td></tr>
<tr><td>ASN 56</td><td>CDR2</td><td>H</td><td>112.21</td><td>21.83 ||</td><td>0.03</td></tr>
<tr><td>THR 57</td><td>CDR2</td><td></td><td>74.73</td><td>3.97 |</td><td>0.01</td></tr>
<tr><td>TYR 58</td><td>CDR2</td><td></td><td>127.07</td><td>101.47 ||||||||</td><td>0.73</td></tr>
<tr><td>PRO 60</td><td>CDR2</td><td></td><td>26.45</td><td>1.84 |</td><td>0.03</td></tr>
<tr><td>PRO 98</td><td>CDR3</td><td></td><td>43.13</td><td>39.61 ||||||||||</td><td>0.63</td></tr>
<tr><td>SER 99</td><td>CDR3</td><td></td><td>71.98</td><td>55.05 ||||||||</td><td>-0.16</td></tr>
<tr><td>ARG 101</td><td>CDR3</td><td>HS</td><td>84.80</td><td>53.72 |||||||</td><td>-1.48</td></tr>
<tr><td>ILE 102</td><td>CDR3</td><td></td><td>138.09</td><td>84.75 |||||||</td><td>1.36</td></tr>
</table>

Polar contacts between hACE2 and VHH B07

hACE2				VHH B07	
**atom**	**SS**	**distance (Å)**		**atom**	**CDR**
HIS 34 NE2	a1	H	3.12	ASN 56 O	CDR2
GLU 35 OE1	a1	HS	3.01	ARG 101 NH1	CDR3
GLU 35 OE2	a1	HS	2.90	ARG 101 NH2	CDR3
GLU 37 OE1	a1	H	3.75	ASN 56 ND2	CDR2
GLU 37 OE1	a1	H	3.40	SER 54 OG	CDR2
ASP 38 OD1	a1	H	2.76	THR 53 OG1	CDR2
ASP 38 OD2	a1	H	2.79	THR 33 OG1	CDR1
ASP 38 OD2	a1	H	3.05	THR 53 N	CDR2
LYS 68 NZ	a2	H	3.26	ASN 32 OD1	CDR1
GLU 75 OE1	a2	HS	3.07	ARG 101 NE	CDR3
GLU 75 OE2	a2	HS	2.89	ARG 101 NH1	CDR3
LYS 353 NZ	loop b4-b5	H	2.79	THR 53 OG1	CDR2

Supplementary Table 4: **Van der Waals and polar interactions between hACE2 and VHH B07**. CDR: complementary-determining region (KABAT definition); FR: Framework region; ss: secondary structure in hACE2; H: hydrogen bonds; S: salt-bridges; ASA: accessible surface area; BSA: buried surface area; ΔGi: solvation energy effect; Vertical bars in BSA columns indicate the buried area percentage (with one bar per 10% of ASA).

<table>
<tr><th colspan="2">hACE2</th><th colspan="2">hACE2/VHH B07-B10 (this work)</th><th colspan="2">hACE2/RBD SARS CoV-2 (PDB 6M0J)</th><th colspan="2">hACE2/RBD SARS CoV (PDB 2AJF)</th><th colspan="2">hACE2/RBD HCoV-NL63 (PDB 3KBH)</th></tr>
<tr><th>residues</th><th>ss</th><th>HS</th><th>BSA (Å^2) VHH B07</th><th>HS</th><th>BSA (Å^2) RBD</th><th>HS</th><th>BSA (Å^2) RBD</th><th>HS</th><th>BSA (Å^2) RBD</th></tr>
<tr><td>SER 19</td><td>N-ter</td><td></td><td></td><td></td><td>10.86 |</td><td></td><td>31.48 |||</td><td></td><td></td></tr>
<tr><td>GLU 23</td><td>α1</td><td></td><td></td><td></td><td></td><td></td><td>1.24 |</td><td></td><td></td></tr>
<tr><td>GLN 24</td><td>α1</td><td></td><td></td><td>H</td><td>54.04 ||||||||</td><td>H</td><td>39.35 |||||</td><td></td><td></td></tr>
<tr><td>**THR 27**</td><td>**α1**</td><td></td><td>**13.25 ||**</td><td></td><td>**67.91 ||||||||**</td><td></td><td>***73.41 ||||||||***</td><td></td><td></td></tr>
<tr><td>PHE 28</td><td>α1</td><td></td><td></td><td></td><td>14.77 ||||||||</td><td></td><td>11.99 |||||||||</td><td></td><td></td></tr>
<tr><td>**ASP 30**</td><td>**α1**</td><td></td><td>**18.60 ||**</td><td>**HS**</td><td>**41.36 ||||||**</td><td></td><td>***13.25 ||***</td><td></td><td>***22.50 |||***</td></tr>
<tr><td>**LYS 31**</td><td>**α1**</td><td></td><td>**48.19 ||||**</td><td></td><td>**91.32 ||||||||**</td><td></td><td>***52.47 |||||||***</td><td></td><td>***3.56 |***</td></tr>
<tr><td>ASN 33</td><td>α1</td><td></td><td></td><td></td><td></td><td></td><td></td><td></td><td>20.55 ||||||||</td></tr>
<tr><td>**HIS 34**</td><td>**α1**</td><td>**H**</td><td>**120.57 |||||||||**</td><td>**H**</td><td>**74.52 ||||||**</td><td></td><td>***62.39 |||||***</td><td></td><td>***61.01 ||||||***</td></tr>
<tr><td>**GLU 35**</td><td>**α1**</td><td>**HS**</td><td>**79.49 ||||||||||**</td><td>**H**</td><td>**14.69 |||**</td><td></td><td></td><td></td><td></td></tr>
<tr><td>**GLU 37**</td><td>**α1**</td><td>**H**</td><td>**46.14 ||||||**</td><td>**H**</td><td>**14.30 ||||**</td><td>***H***</td><td>***17.13 |||||***</td><td>***H***</td><td>***37.61 |||||||||***</td></tr>
<tr><td>**ASP 38**</td><td>**α1**</td><td>**H**</td><td>**77.51 ||||||||||**</td><td>**H**</td><td>**32.56 ||||||**</td><td>***H***</td><td>***34.28 ||||||***</td><td></td><td>***1.97 |***</td></tr>
<tr><td>LEU 39</td><td>α1</td><td></td><td>33.13 ||||||||</td><td></td><td></td><td>H</td><td></td><td></td><td></td></tr>
<tr><td>TYR 41</td><td>α1</td><td></td><td></td><td>H</td><td>42.44 |||||||||||</td><td>H</td><td>44.99 ||||||||||</td><td>H</td><td>35.07 |||||||</td></tr>
<tr><td>**GLN 42**</td><td>**α1**</td><td></td><td>**48.95 ||||||**</td><td>**H**</td><td>**46.41 ||||||**</td><td>***H***</td><td>***32.19 ||||***</td><td></td><td></td></tr>
<tr><td>LEU 45</td><td>α1</td><td></td><td></td><td></td><td>27.84 ||||||</td><td></td><td>33.41 ||||||</td><td></td><td></td></tr>
<tr><td>LYS 68</td><td>α2</td><td>H</td><td>51.24 |||||</td><td></td><td></td><td></td><td></td><td></td><td></td></tr>
<tr><td>ALA 71</td><td>α2</td><td></td><td>25.40 |||||</td><td></td><td></td><td></td><td></td><td></td><td></td></tr>
<tr><td>PHE 72</td><td>α2</td><td></td><td>14.52 ||||||||||</td><td></td><td></td><td></td><td></td><td></td><td></td></tr>
<tr><td>GLU 75</td><td>α2</td><td>HS</td><td>54.58 ||||||</td><td></td><td></td><td></td><td></td><td></td><td></td></tr>
<tr><td>GLN 76</td><td>α2</td><td></td><td>2.04 |||</td><td></td><td></td><td></td><td></td><td></td><td></td></tr>
<tr><td>LEU 79</td><td>α2</td><td></td><td></td><td></td><td>25.95 |||||</td><td></td><td>31.12 ||||</td><td></td><td></td></tr>
<tr><td>MET 82</td><td>α2-η1</td><td></td><td></td><td></td><td>27.86 |||</td><td></td><td>37.04 |||</td><td></td><td></td></tr>
<tr><td>TYR 83</td><td>α2-η1</td><td></td><td></td><td>H</td><td>36.50 ||||||||||</td><td>H</td><td>31.15 |||||||</td><td></td><td></td></tr>
<tr><td>PRO 321</td><td>α12-α13</td><td></td><td></td><td></td><td></td><td></td><td></td><td></td><td>19.46 |||</td></tr>
<tr><td>ASN 322</td><td>α12-α13</td><td></td><td></td><td></td><td></td><td></td><td></td><td></td><td>12.31 ||</td></tr>
<tr><td>MET 323</td><td>α12-α13</td><td></td><td></td><td></td><td></td><td></td><td></td><td></td><td>1.96 ||</td></tr>
<tr><td>THR 324</td><td>α12-α13</td><td></td><td></td><td></td><td>2.18 |</td><td></td><td>5.02 ||</td><td>H</td><td>44.17 ||||||||||</td></tr>
<tr><td>GLN 325</td><td>α13</td><td></td><td></td><td></td><td>8.02 |</td><td>H</td><td>43.00 ||||</td><td></td><td>42.15 ||||</td></tr>
<tr><td>GLY 326</td><td>α13</td><td></td><td></td><td></td><td>2.01 |</td><td></td><td>13.88 |||||||</td><td></td><td>19.16 ||||||||||</td></tr>
<tr><td>GLU 329</td><td>α13</td><td></td><td></td><td></td><td></td><td>HS</td><td>27.64 |||</td><td></td><td></td></tr>
<tr><td>ASN 330</td><td>α13</td><td></td><td></td><td></td><td>28.75 |||||</td><td>H</td><td>33.10 ||||||</td><td></td><td>15.67 ||</td></tr>
<tr><td>**LYS 353**</td><td>**loop** *β4-β5*</td><td>**H**</td><td>**28.47 |||**</td><td>**H**</td><td>**98.99 |||||||||**</td><td>***H***</td><td>***104.54 ||||||||||***</td><td>***H***</td><td>***65.15 |||||||***</td></tr>
<tr><td>GLY 354</td><td>β5</td><td></td><td></td><td></td><td>30.22 ||||||</td><td></td><td>27.83 ||||||</td><td></td><td>54.04 ||||||||||</td></tr>
<tr><td>ASP 355</td><td>β5</td><td></td><td></td><td></td><td>12.29 ||||||||</td><td></td><td>10.29 |||||||||</td><td></td><td>15.47 ||||||||||</td></tr>
<tr><td>PHE 356</td><td>β5</td><td></td><td></td><td></td><td></td><td></td><td></td><td></td><td>10.01 |||||||||</td></tr>
<tr><td>ARG 357</td><td>β5</td><td></td><td></td><td></td><td>12.01 |||||||</td><td></td><td>10.82 |||||||</td><td></td><td>3.79 |||</td></tr>
<tr><td>MET 383</td><td>α14</td><td></td><td></td><td></td><td></td><td></td><td></td><td></td><td>30.27 |||||||</td></tr>
<tr><td>ALA 384</td><td>α14</td><td></td><td></td><td></td><td></td><td></td><td></td><td></td><td>1.90 ||</td></tr>
<tr><td>ALA 386</td><td>α14</td><td></td><td></td><td></td><td>0.25 |</td><td></td><td></td><td></td><td>23.96 ||||||||</td></tr>
<tr><td>ALA 387</td><td>α14</td><td></td><td></td><td></td><td></td><td></td><td></td><td>H</td><td>40.36 |||||</td></tr>
<tr><td>PRO 390</td><td>η7</td><td></td><td></td><td></td><td></td><td></td><td></td><td></td><td>1.57 |</td></tr>
<tr><td>ARG 393</td><td>η7-α15</td><td></td><td></td><td>H</td><td>10.70 ||</td><td></td><td>6.70 ||</td><td></td><td>23.51 |||||</td></tr>
<tr><td>PHE 555</td><td>α22</td><td></td><td></td><td></td><td></td><td></td><td></td><td></td><td>10.92 ||</td></tr>
</table>

Supplementary Table 5: **Van der Waals contacts between hACE2 and VHH B07 and between hACE2 and RBDs for a selection of viruses using hACE2 as a receptor.** ss: secondary structure of hACE2; H: hydrogen bonds; S: salt-bridges; BSA: buried surface area; Vertical bars in BSA columns indicate the buried area percentage (with one bar per 10% of ASA of each indicated residue). In bold red are indicated the common interactions between B07 and SARS-CoV-2 RBD structures. In bold red italic are indicated the common interactions between the four structures.
