# Supplementary material for: Targeting ACE2 with a camelid antibody inhibits SARS-CoV-2 binding and has protective effects in vivo
Source: Nat Commun. 2025 Nov 21;16:10268. doi: 10.1038/s41467-025-65144-w (PMC12638815; doi:10.1038/s41467-025-65144-w)
Supplement: Supplementary file 2 — Reporting Summary [file 41467_2025_65144_MOESM2_ESM.pdf]

Corresponding author(s): Anne Brelot
Last updated by author(s): Sep 25, 2025

Reporting Summary

Nature Portfolio wishes to improve the reproducibility of the work that we publish. This form provides structure for consistency and transparency in reporting. For further information on Nature Portfolio policies, see our Editorial Policies and the Editorial Policy Checklist.

Statistics

For all statistical analyses, confirm that the following items are present in the figure legend, table legend, main text, or Methods section.

n/a	Confirmed	
☐	☒	The exact sample size (*n*) for each experimental group/condition, given as a discrete number and unit of measurement
☐	☒	A statement on whether measurements were taken from distinct samples or whether the same sample was measured repeatedly
☐	☒	The statistical test(s) used AND whether they are one- or two-sided<br>*Only common tests should be described solely by name; describe more complex techniques in the Methods section.*
☒	☐	A description of all covariates tested
☒	☐	A description of any assumptions or corrections, such as tests of normality and adjustment for multiple comparisons
☐	☒	A full description of the statistical parameters including central tendency (e.g. means) or other basic estimates (e.g. regression coefficient) AND variation (e.g. standard deviation) or associated estimates of uncertainty (e.g. confidence intervals)
☐	☒	For null hypothesis testing, the test statistic (e.g. *F*, *t*, *r*) with confidence intervals, effect sizes, degrees of freedom and *P* value noted<br>*Give P values as exact values whenever suitable.*
☒	☐	For Bayesian analysis, information on the choice of priors and Markov chain Monte Carlo settings
☒	☐	For hierarchical and complex designs, identification of the appropriate level for tests and full reporting of outcomes
☒	☐	Estimates of effect sizes (e.g. Cohen's *d*, Pearson's *r*), indicating how they were calculated

Our web collection on statistics for biologists contains articles on many of the points above.

Software and code

Policy information about availability of computer code

Data collection	Attune Nxt Software v3.2.1 (ThermoFisher), Octet Data Analysis Studio, v13.0 (Sartorius), Harmony Software v4.9 (PerkinElmer), Tecan Spark Software SparkControl Magellan 3.2, QuantStudio Software v2.6.0 (Applied Biosystems), Berthold Software (mikroWin 2000), XFLUOR4 v4.51,ZEN software 2012 (Zeiss), X-ray data processing : XDS v20241002; X-ray data scaling and merging : AIMLESS from CCP4 interface v9.0.008; X-ray data phasing: PHASER v2.8.3; X-ray data refinement : BUSTER/TNT v20240710; Structure modeling : COOT v0.9.8.96; Structure validation : MOLPROBITY (http://molprobity.biochem.duke.edu/) v4.5.2; Structure analysis : PISA (https://www.ebi.ac.uk/msd-srv/prot_int/pistart.html) v1.48 ; Structure display : PyMol Molecular Graphics System 3.0.3.
Data analysis	Prism v10.2.3 (Graph Pad Software), Excel 365v2311 (Microsoft), Kaluza v2.1 windows 10 (Beckman Coulter), SnapGene v8.1.0 (GSL Biotech LLC), ImageJ v2.16.0 (Fiji), Clustal Omega https://www.ebi.ac.uk › clustalo

For manuscripts utilizing custom algorithms or software that are central to the research but not yet described in published literature, software must be made available to editors and reviewers. We strongly encourage code deposition in a community repository (e.g. GitHub). See the Nature Portfolio guidelines for submitting code & software for further information.

Data

Policy information about availability of data

All manuscripts must include a data availability statement. This statement should provide the following information, where applicable:

- Accession codes, unique identifiers, or web links for publicly available datasets
- A description of any restrictions on data availability
- For clinical datasets or third party data, please ensure that the statement adheres to our policy

All data supporting the results of this study can be found in this article, the Supplementary Information, and the Source data file. Coordinates and structure factors have been deposited in the Protein Data Bank under the accession code 9R19[https://doi.org/10.2210/pdb9R19/pdb] (https://www.rcsb.org/structure/9R19). The unique materials used in this study are readily available from the authors upon request (Materials Transfer Agreement with the Institut Paseur) for academic research purposes. Source data are provided with this paper.

Research involving human participants, their data, or biological material

Policy information about studies with human participants or human data. See also policy information about sex, gender (identity/presentation), and sexual orientation and race, ethnicity and racism.

Reporting on sex and gender	NA
Reporting on race, ethnicity, or other socially relevant groupings	NA
Population characteristics	NA
Recruitment	NA
Ethics oversight	NA

Note that full information on the approval of the study protocol must also be provided in the manuscript.

Field-specific reporting

Please select the one below that is the best fit for your research. If you are not sure, read the appropriate sections before making your selection.

[x] Life sciences [ ] Behavioural & social sciences [ ] Ecological, evolutionary & environmental sciences

For a reference copy of the document with all sections, see nature.com/documents/nr-reporting-summary-flat.pdf

Life sciences study design

All studies must disclose on these points even when the disclosure is negative.

Sample size	The sample size for in vitro experiments is indicated in the figure legends and method section. Sample size for mouse and hamster studies were determined based upon previous experience with similar studies where 4-5 animals per group represented a sufficient sample size to detect statistical differences between experimental groups regarding viral load, while 8-10 were needed to compare survival rates. Sample size was increased to confirm a non-significant trend identified in the first group of animals.
Data exclusions	None
Replication	All experiments were performed and verified in multiple replicates as indicated in figure legends.<br>Mouse experiments were repeated 2 times.
Randomization	The Syrian golden hamsters were each randomly allocated to two experimental groups. Mice were allocated to experimental groups to balance sex and age.
Blinding	The investigators were blinded to the studies involving mice and hamsters. Each experimental group was assigned a number prior to dissection and analysis, ensuring that subsequent procedures were conducted as single-blind experiments.<br>The student performing the ELISA assays to measure the dose of VHH present in the lung homogenate was blinded to group association and samples were analyzed without knowledge of their origin. The same procedure was applied for the engineer who performed immunofluorescence experiments on mouse organs.

Reporting for specific materials, systems and methods

We require information from authors about some types of materials, experimental systems and methods used in many studies. Here, indicate whether each material, system or method listed is relevant to your study. If you are not sure if a list item applies to your research, read the appropriate section before selecting a response.

Materials & experimental systems

n/a	Involved in the study	
	x	Antibodies
	x	Eukaryotic cell lines
x		Palaeontology and archaeology
	x	Animals and other organisms
x		Clinical data
x		Dual use research of concern
x		Plants

Methods

n/a	Involved in the study	
x		ChIP-seq
	x	Flow cytometry
x		MRI-based neuroimaging

Antibodies

Antibodies used

- Anti-M13 Antibody, Mouse Monoclonal from Sinobiological (cat# 11973-MM05T)
- SARS-CoV-2 nucleocapsid mouse antibody (GeneTex, GTX36802, clone 3851, dilution 1:100)
- Spike monoclonal antibody mAb48 (kind gift of H. Mouquet, Institut Pasteur)
- Anti-ACE2 VHH B07 (10 µg/mL)(Flow cytometry) (this article)
- Anti-ACE2 VHH B09 (10 µg/mL)(Flow cytometry) (this article)
- Anti-ACE2 VHH B10 (10 µg/mL)(Flow cytometry) (this article)
- Anti-ACE2 VHH B07-Fc (0.1 to 6 µg/mL) (Flow cytometry, Microscopy) (this article)
- VHH control: anti-IgE, anti-IgM, VHH-Fc ctl (this article)
- Mouse monoclonal Myc-tag 9E10 (Abnova, Cat# MAB0967, lot 543989, dilution 1 :500)
- Rat anti-alpha tubulin (ThermoFisher; cat# MA1-80017; clone YL1/2, dilution 1:200)

- Goat anti-mouse IgG Alexa Fluor 488 (Invitrogen; cat# A11029, lot 2486523, dilution 1:200)
- Goat anti-mouse IgG Alexa Fluor 647 (Invitrogen; cat# A21236, lot 2674387, dilution 1:200)
- Goat anti-mouse IgG Alexa Fluor 555 (Abcam; cat# ab150114, dilution 1:200)
- Goat anti-human IgG Alexa Fluor488 (ThermoFischer Cat# A11013, lot 2991659, dilution, 1:200)
- Goat anti-human IgG Alexa Fluor647 (ThermoFischer Cat# A21445, lot 2941304, dilution, 1:200)
- Goat anti-human AlexaFluor 594 (Jackson immuno Research, Cat# 109-587-003, dilution 1:200)
- Donkey anti-rat Alexa-Fluor 488 (Invitrogen Cat# A-21208, dilution 1:200)
- Phalloidin-Atto 647 (Sigma, Cat# 65906, dilution 1:400)
- Peroxydase affinitipure rabbit anti-hman IgG (Jackson immuno Research, Cat# AB_2339647, dilution 1:1000)

Validation

The reactivity of VHHs to ACE2 was validated using ELISA binding assay, BLI, Flow cytometry (this article).
Validation of mAb 48 has been performed by H. Mouquet (ref 67).
All other antibodies used in this study are commercially available and have been validated by the manufacturers and described in other publications: anti-myc 9E10: https://www.abnova.com/en-global/product/detail/MAB0967 ; anti-M13: https://www.sinobiological.com/antibodies/m13-11973-mm05t; anti-N: https://www.genetex.com/Product/Detail/SARS-CoV-Nucleoprotein-antibody-3851/GTX36802?srsltid=AfmBOoqEvNKkYemlPr8_mYkf69XXNy6ETbZKqzrn2IfBgjVZ2rnMdpd4; anti-tubulin: https://www.thermofisher.com/antibody/product/alpha-Tubulin-Antibody-clone-YL1-2-Monoclonal/MA1-80017

Eukaryotic cell lines

Policy information about cell lines and Sex and Gender in Research

Cell line source(s)

Expi293FTM (ThermoFisher, ref A14527), HEK293 (ATCC CLR 1573), IGROV1 (NCI-60), Vero E6 (ATCC Cat#CRL-1586), A549 (ATCC cat# CCL-185), U2OS (ATCC Cat# HTB-96). U2OS-GFP1-10 and 11 (S-Fuse cells) were derived from U2OS (ref. 32).

Authentication

Cell lines from ATCC or ThermoFisher were not indepentdently authenticated. U2OS-derived cells has been authenticated by genotyping (Eurofins).

Mycoplasma contamination

All cells are negative for mycoplasma contamination.

Commonly misidentified lines
(See ICLAC register)

None

Animals and other research organisms

Policy information about studies involving animals; ARRIVE guidelines recommended for reporting animal research, and Sex and Gender in Research

Laboratory animals

- Alpaca: Lama pacos, male, 7 years old
- B6.Cg-Tg(K18-ACE2)2Prlmn/J mice (stock #034860) were imported from The Jackson Laboratory, 10 to 13 wk-old male and female mice.
- Golden Syrian hamsters (Mesocricetus auratus, strain RjHan:AURA), aged 6 week

Wild animals	The study did not involve wild animals.
Reporting on sex	Syrian golden hamsters used in this study were male, while K18-hACE2 mice included both male (13) and female (12) subjects. The sex of the animals was not considered a variable in this study.
Field-collected samples	The study did not involve sample collection from the field.
Ethics oversight	Alpaca: All immunization processes were executed according to the French legislation and in compliance with the European Communities Council Directives (2010/63/UE, French Law 2013-118, February 6, 2013). The Animal Experimentation Ethics Committee of Pasteur Institute (CETEA 89) approved this study (2020-27412).<br><br>K18-hACE2: All animals were handled in strict accordance with good animal practice. Animal work was approved by the Animal Experimentation Ethics Committee (CETEA 89) of the Institut Pasteur (project dap 210050), and authorized by the French Ministry of Research (under project 31816) before the experiments were initiated.<br><br>Golden hamsters were housed and manipulated in class III safety cabinets in the Institut Pasteur animal facilities accredited by the French Ministry of Agriculture. Animal work was approved by the Animal Experimentation Ethics Committee (CETEA) of the Institut Pasteur (project dap 210011) and authorized by the French legislation (project #21045) in compliance with the European Communities Council Directives (2010/63/UE, French Law 2013-118, February 6, 2013) and according to the regulations of Institut Pasteur Animal Care Committees before the experiments were initiated.

Note that full information on the approval of the study protocol must also be provided in the manuscript.

Plants

Seed stocks	NA
Novel plant genotypes	NA
Authentication	NA

Flow Cytometry

Plots

Confirm that:

- [x] The axis labels state the marker and fluorochrome used (e.g. CD4-FITC).
- [x] The axis scales are clearly visible. Include numbers along axes only for bottom left plot of group (a 'group' is an analysis of identical markers).
- [x] All plots are contour plots with outliers or pseudocolor plots.
- [x] A numerical value for number of cells or percentage (with statistics) is provided.

Methodology

Sample preparation	Cells were stained as indicated in the method section. All samples were acquired within 24-48h.
Instrument	Attune Nxt Flow Cytometer (Invitrogen catalog number : 15360667)
Software	Attune NxT Software v3.2.1 for acquisition, Kaluza Software for analysis
Cell population abundance	At least 10,000 cells were acquired for each condition.
Gating strategy	All gates were set on unstained cells and, if applicable, on GFP positive cells.

- [x] Tick this box to confirm that a figure exemplifying the gating strategy is provided in the Supplementary Information.
